# Parameterization and reliability of single-leg balance test assessed with inertial sensors in stroke survivors: a cross-sectional study

**DOI:** 10.1186/1475-925X-13-127

**Published:** 2014-08-30

**Authors:** David Perez-Cruzado, Manuel González-Sánchez, Antonio Ignacio Cuesta-Vargas

**Affiliations:** Departamento de Psiquiatria y Fisioterapia, Instituto de Investigación Biomédica de Málaga (IBIMA), Universidad de Málaga, 29071 Málaga, Spain; School of Clinical Sciences of the Faculty of Health, Queensland University of Technology, Brisbane, Australia

**Keywords:** Reliability, Inertial sensor, Stroke, Single-leg stance test, Balance

## Abstract

**Background and purpose:**

There are no published studies on the parameterisation and reliability of the single-leg stance (SLS) test with inertial sensors in stroke patients. Purpose: to analyse the reliability (intra-observer/inter-observer) and sensitivity of inertial sensors used for the SLS test in stroke patients. Secondary objective: to compare the records of the two inertial sensors (trunk and lumbar) to detect any significant differences in the kinematic data obtained in the SLS test.

**Methods:**

Design: cross-sectional study. While performing the SLS test, two inertial sensors were placed at lumbar (L_5_-S_1_) and trunk regions (T_7_–T_8_). Setting: Laboratory of Biomechanics (Health Science Faculty - University of Málaga). Participants: Four chronic stroke survivors (over 65 yrs old). Measurement: displacement and velocity, Rotation (X-axis), Flexion/Extension (Y-axis), Inclination (Z-axis); Resultant displacement and velocity (V):

Along with SLS kinematic variables, descriptive analyses, differences between sensors locations and intra-observer and inter-observer reliability were also calculated.

**Results:**

Differences between the sensors were significant only for left inclination velocity (p = 0.036) and extension displacement in the non-affected leg with eyes open (p = 0.038). Intra-observer reliability of the trunk sensor ranged from 0.889-0.921 for the displacement and 0.849-0.892 for velocity. Intra-observer reliability of the lumbar sensor was between 0.896-0.949 for the displacement and 0.873-0.894 for velocity. Inter-observer reliability of the trunk sensor was between 0.878-0.917 for the displacement and 0.847-0.884 for velocity. Inter-observer reliability of the lumbar sensor ranged from 0.870-0.940 for the displacement and 0.863-0.884 for velocity.

**Conclusion:**

There were no significant differences between the kinematic records made by an inertial sensor during the development of the SLS testing between two inertial sensors placed in the lumbar and thoracic regions. In addition, inertial sensors. Have the potential to be reliable, valid and sensitive instruments for kinematic measurements during SLS testing but further research is needed.

## Introduction

Stroke is the most common cause of severe disability and the third-leading cause of death in the western world which increases with age [1]. Imbalance is one of the major resulting symptoms of stroke survivors [2]. Imbalance is defined as body instability (static and dynamic) [2]. It increases the difficulty of daily activities for stroke patients and thus reduces their independence [3].

Within the clinic, diverse functional tests are used to measure the balancing capability of subjects [4, 5]. A balance test, known as the single-leg stance test (SLS), is widely used for diagnosis and monitoring of patients in research and clinical settings. Owing to its simplicity, high-reliability and low cost [6–8]. SLS tests have been used to assess balance and postural control in patients that have had a stroke [9–11]. However, the main result of this test (seconds) is greatly enriched if supplemented with kinematic records, which analyse objectively the subject movement during the test. Inertial sensors are frequently-used instruments for kinematic analysis of positions and movements [3, 12, 13]. Inertial sensors which are portable, non-invasive, highly-accurate instruments and without side-effects can be used to measure the kinematic parameters of gestures [12, 13]. They have a range of validity of 0.657–0.998 [12] and reliability of 0.84–0.97 [3]. These features have favoured its used in basic and clinical research.

The parameterisation of simple functional tests via the insertion of instruments into everyday devices such as smartphones and watches, could have enormous clinical potential in the diagnosis, assessment and monitoring of patients with chronic diseases [12, 14, 15].

Some common locations for placing the inertial sensors are: the centre of mass [16, 17], or trunk [18–20]. However, there are no published studies analysing which of the two places is best for kinematic recording during SLS testing. Furthermore, no study has been found that analyses the reliability of parameterisation of SLS testing in stroke patients.

The main objective of this study was to analyse whether there are significant differences in kinematic records between two inertial sensors (one positioned on the trunk and another placed in the lumbar region) during an SLS test. In addition, a second objective of the study was to analyse the reliability (intra-observer/inter-observer reliability) and sensitivity of inertial sensors used for the SLS test and to specify parameters for the SLS test when it is conducted using inertial sensors in stroke patients. We hypothesised that there would be significant differences between kinematic data from the two different sensor locations (trunk and lumbar region) during the SLS test. Moreover, inertial sensors would prove to be a reliable tool when used for the kinematic analysis of postural control in stroke patients.

## Methods

### Design and participants

This study was a cross-sectional study that involved four participants that had previously had a stroke. Inclusion criteria for participants were: age ≥65 years; more than 6 months post diagnosis of stroke; capable of walking at least 15 m without the aid of a walker; capable of following verbal instructions and able to stand for >30 seconds without assistance. Exclusion criteria for participants were: people with severe hemi-neglect; history or current diagnosis of other neurological or musculoskeletal impairment or pain. No racial/ethnic-based differences were present.

Ethical approval for the study was granted by the Ethics Committee of the Faculty of Health Sciences, University of Malaga. The study complied with the principles laid out in the Declaration of Helsinki.

Before the SLS test, each participant was given an information sheet and provided informed consent for participation. Participants were informed that participation was voluntary and they could withdraw at any point. They were also assured that their personal data would be treated in accordance with the Organic Law of Protection of Personal Data.

### Inertial sensors

The inertial sensors (IS) used in this study were the InertiaCube3 model (InterSense Inc., Bedford, MA, USA) with a sampling frequency of 180 Hz. The InertiaCube3 is a small sensor (26.2 mm × 39.2 mm × 14.8 mm), based on micro-electro-mechanical systems (MEMS) technology and does not incorporate castors, which might generate noise, inertial forces and increase the risk of mechanical failure. The InertiaCube3 measures nine physical properties simultaneously, namely: angular rates, linear accelerations and magnetic field components along the three axes (yaw, pitch and roll). Miniature vibrating elements are used to measure all angular velocities and linear accelerations.

**Figure 1:**
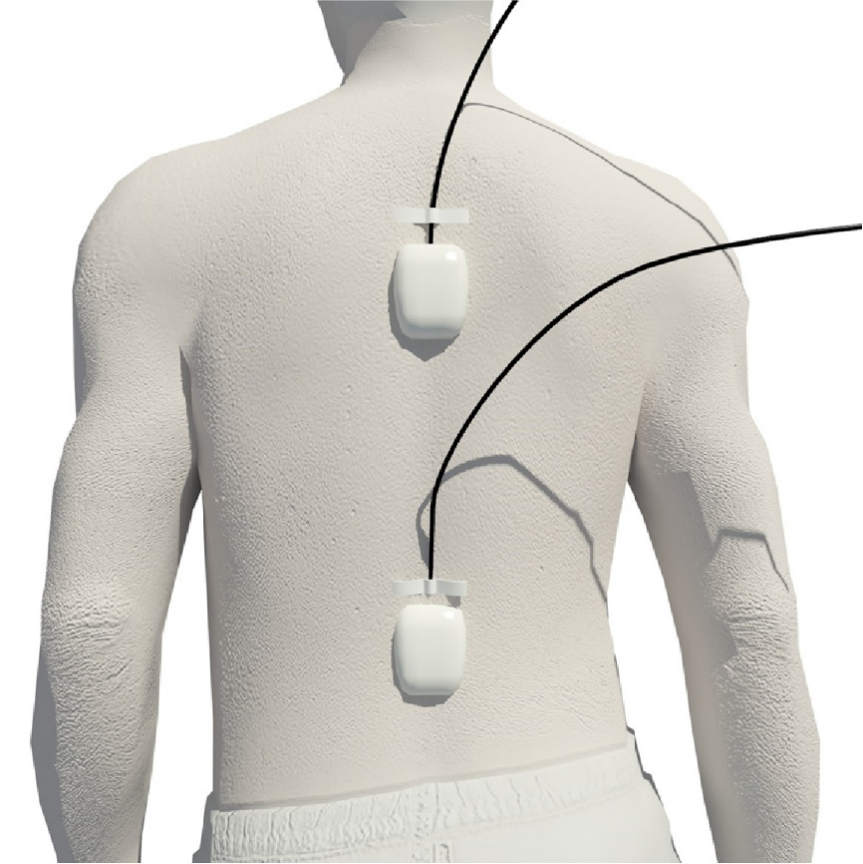
**Localization of the inertial sensors.**

Two inertial sensors were placed in the trunk (T_7_– T_8_) and in lumbar (L_5_–S_1_) regions to collect kinematic data whilst the patient was performing the test (Figure 1). The IS were fixed to the skin using double adhesive and reinforced with inextensible tape surrounding the entire diameter of the trunk or the lumbar region of each participant. Movement recording started 3 s before the patient started the SLS and finished 3 s after the patient had completed the test to allow the identification of the start and the end of the test in the kinematic record.

**Figure 2:**
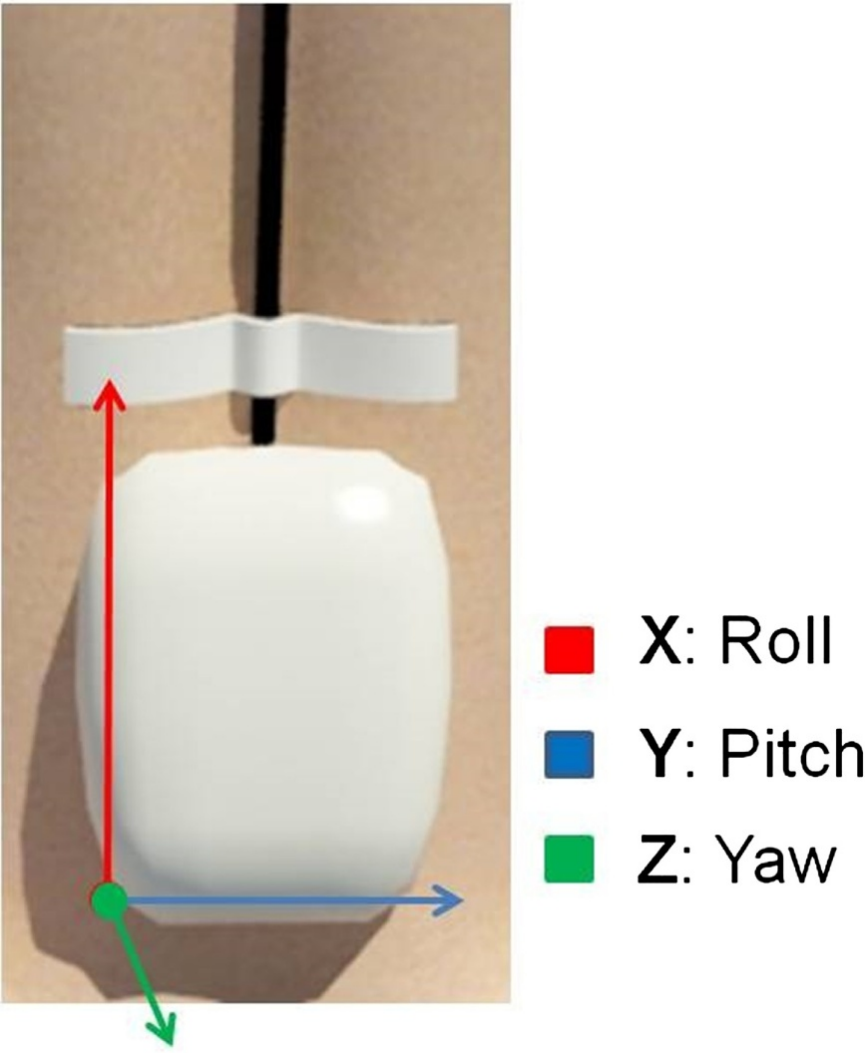
**Origin of the coordinates in the inertial sensors.**

Sensors were positioned so that the origin of the coordinates was in the postero-inferior left corner (Figure 2).

**Figure 3:**
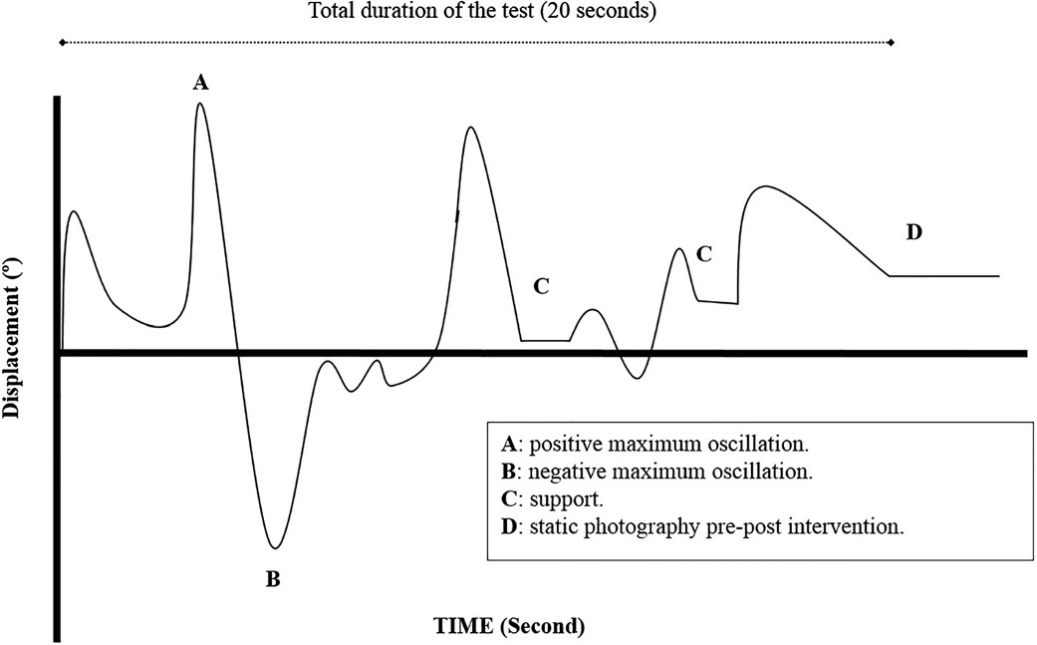
**Example of graphs generated during the SLS.**

After data collection had been completed we extracted kinematic data (direct and indirect) offline from all the graphs generated during the SLS test by all of the participants (Figure 3).

### Procedure: single-leg stance (SLS) test

To perform the SLS test, each participant must remain supported on one leg, arms resting on the hips. The test is timed (in seconds) from the moment the participant gets into the test position, until the other foot touches the ground or until the arms are separated from the hips [21].

Slight modifications to the protocol were made to standardise the implementation of the test for all participants. To ensure the safety of participants (who all had a diminished sense of balance) two researchers stood in front of and behind each patient to ensure safety in the event of serious instability which might result in a fall. Although the patient stood on the floor, he or she was surrounded by mats to minimise the negative effects of any fall. The time record began when the opposite foot of leg support was lifted from the floor until the foot touched the other leg or the ground, losing the balance [22, 23]. At the start of the test the patient was instructed to lift one leg with a knee flexion of 90 degrees, and to retain this static single-leg stance for 30 seconds [22–24].

The SLS test was carried out three times for each condition and in the following order: non-affected leg, eyes open; non-affected leg, eyes closed; affected leg, eyes open and affected leg, eyes closed. The resting time between each repetition was 150 seconds and they were allowed to sit during rest period. Patients were given the opportunity to practise as many times as they wanted to in order to make sure they understood how to perform the SLS test.

Before the test, patients stood in a relaxed position on two legs. We explained and demonstrated the test to help patients understand what was required of them. It has been reported that the reliability of the SLS test is 0.89 and 0.86 with eyes opened and closed, respectively, in elderly people [25].

Each participant performed the same protocol twice on two different days. The raters that supervised the protocols were different on the first and second day.

### Variables

The variables measured in the present study were 136 variables in total (72 displacement variables and 64 velocity variables); 9 displacement variables in each condition (4 conditions) in each inertial sensor (lumbar and trunk) and 8 velocity variables in each condition (4 condition) in each inertial sensor (lumbar and trunk).

**Displacement:** Rotation (left/right side): maximum positive displacement (right) and negative displacement (left) relative to the X-axis. Flexion/extension: maximum positive displacement (flexion) and negative displacement (extension) relative to the Y-axis. Inclination (left/right side): maximum positive displacement (right) and negative (left) relative to the Z-axis. Mean displacement: mean displacement of each axis (X, Y, Z). **Velocity:** Rotation (left/right side): maximum positive velocity (right) and negative velocity (left) relative to the X-axis. Flexion/Extension: maximum positive velocity (flexion) and negative velocity (extension) relative to the Y-axis. Inclination (left/right side): maximum positive velocity (right) and negative velocity (left) relative to the Z-axis. Resultant velocity: the resultant velocity (V) vector was calculated using the formula:


### Data analysis

We analysed anthropometric measurements and the data obtained from various self-report questionnaires designed for use in patients with neurological impairments. We also made a descriptive analysis of the kinematic data from the two inertial sensors (trunk and lumbar).

The Kolmogorov-Smirnov (K-S) test was used to assess the distribution of kinematic data. Following this we compared data from the trunk and lumbar sensors the normally distributed data was analysed using the parametric student *t*-test and non-normally distributed data was analysed using the non-parametric Wilcoxon’s test. A significance threshold of p ≤ 0.05 was used.

Reliability measures were calculated by analysing the internal consistency (intra-class correlation coefficients (ICC 3,1) were calculated for intra-observer and inter-observer reliability) of the measures with 95% confidence intervals for each outcome variable. Reliability was classified as follows: excellent (ICC > 0.80), good (0.80 > ICC > 0.60), moderate (0.60 > ICC > 0 .40), or poor (ICC < 0.40) [26].

Data analysis was performed with SPSS (version17.0 for Windows; SPSS Inc., Illinois, and USA).

## Results

The sample was 4 participants. The mean age of the participants was 76.7 (±3.44) years. The values of the various functional tests that the participants completed are also given. These were intended to identify the degree of balance impairment suffered by the participants as a consequence of their stroke (Canadian Neurological Scale: 8.5 ± 0.41; Barthel index 92.5 ± 6.46 and Stroke Impact Scale-16, 67 ± 7.83).

The ICCs were higher than 0.847 (95% CI: 0.836–0.860) for all variables. Intra-observer reliability of the trunk sensor ranged from 0.889-0.921 for the displacement and 0.849-0.892 for velocity. Intra-observer reliability of the lumbar sensor was between 0.896-0.949 for the displacement and 0.873-0.894 for velocity. Inter-observer reliability of trunk sensor was between 0.878-0.917 for the displacement and 0.847-0.884 for velocity. Inter-observer reliability of the lumbar sensor ranged from 0.870-0.940 for the displacement and 0.863-0.884 for velocity. Other values for reliability of the variables are presented in Table 1.

**Table 1:** **Intra-observer and inter-observer reliability of variables measured directly during single leg stance test (SLS)**

	Trunk						Lumbar					
	Intra-observer			Inter-observer			Intra-observer			Inter-observer		
Variable	ICC	IC (95%)		ICC	IC (95%)		ICC	IC (95%)		ICC	IC (95%)	
		Min.	Max.		Min.	Max.		Min.	Max.		Min.	Max.
Left rotation Displacement (°)	**0.912**	0.891	0.928	**0.908**	0.887	0.920	**0.927**	0.905	0.948	**0.915**	0.901	0.927
Right rotation Displacement (°)	**0.903**	0.882	0.914	**0.900**	0.892	0.911	**0.922**	0.910	0.933	**0.909**	0.900	0.917
Rotation Mean Displacement (°)	**0.921**	0.907	0.931	**0.917**	0.907	0.926	**0.937**	0.920	0.955	**0.928**	0.909	0.941
Flexion Displacement (°)	**0.889**	0.870	0.903	**0.879**	0.840	0.901	**0.897**	0.879	0.915	**0.889**	0.878	0.903
Extension Displacement (°)	**0.902**	0.884	0.919	**0.892**	0.883	0.900	**0.913**	0.896	0.930	**0.903**	0.892	0.918
F/E mean Displacement (°)	**0.893**	0.880	0.899	**0.878**	0.867	0.899	**0.896**	0.886	0.908	**0.870**	0.861	0.884
Right inclination Displacement (°)	**0.908**	0.901	0.925	**0.901**	0.886	0.908	**0.933**	0.929	0.941	**0.926**	0.909	0.941
Left inclination Displacement (°)	**0.915**	0.902	9.31	**0.903**	0.891	0.923	**0.949**	0.923	0.960	**0.940**	0.927	0.958
R/L mean inclination Displacement (°)	**0.909**	0.898	0.927	**0.898**	0.883	0.910	**0.928**	0.910	0.940	**0.907**	0.896	0.919
Left rotation Velocity (°/s)	**0.873**	0.865	0.880	**0.862**	0.854	0.879	**0.892**	0.879	0.909	**0.884**	0.868	0.911
Right rotation Velocity (°/s)	**0.863**	0.852	0.870	**0.860**	0.849	0.874	**0.888**	0.873	0.901	**0.880**	0.870	0.891
Flexion Velocity (°/s)	**0.881**	0.870	0.902	**0.874**	0.861	0.886	**0.879**	0.863	0.892	**0.863**	0.852	0.871
Extension Velocity (°/s)	**0.892**	0.881	0.903	**0.884**	0.870	0.896	**0.890**	0.881	0.903	**0.882**	0.870	0.903
Right inclination Velocity (°/s)	**0.855**	0.843	0.867	**0.849**	0.831	0.862	**0.873**	0.861	0.887	**0.869**	0.855	0.878
Left inclination Velocity (°/s)	**0.849**	0.840	0.861	**0.847**	0.836	0.860	**0.881**	0.869	0.897	**0.879**	0.863	0.892
Resultant Velocity Right Side (°/s)	**0.860**	0.848	0.871	**0.857**	0.849	0.867	**0.890**	0.882	0.905	**0.879**	0.866	0.895
Resultant Velocity Left Side (°/s)	**0.873**	0.865	0.884	**0.869**	0.860	0.880	**0.894**	0.876	0.911	**0.882**	0.870	0.899

Tables 2 and 3 show mean values for displacement and velocity. Results are shown as a function of the sensor location (trunk or lumbar), supporting leg (affected or non-affected leg) and test performed (SLS test, eyes open: Table 2; SLS test, eyes closed: Table 3). The tables show differences between inertial sensors. There were significant differences in leftward inclination velocity (p = 0.036) and extension displacement for the non-affected leg (p = 0.038), eyes open condition.

**Table 2:** **Mean values of the kinematic variables during one single leg stance test (eyes open)**

	Non-affected leg				Affected leg			
	Lumbar (sd)	Trunk (sd)	Difference (sd)	p value	Lumbar (sd)	Trunk (sd)	Difference (sd)	p value
Left rotation Displacement^a^ (°)	**13.74** (±12.54)	**5.92** (±2.00)	**7.82** (±7.33)	**0.352**	**11.54** (±11.90)	**7.52** (±7.97)	**4.03** (±8.02)	**0.421**
Right rotation Displacement^a^ (°)	**2.72** (±4.75)	**−0.79** (±3.06)	**−1.94** (±3.26)	**0.067**	**21.44** (±21.23)	**13.24** (±3.81)	**−8.20**(±12.69)	**0.276**
Rotation Mean Displacement^a^ (°)	**7.70**_**L**_	**46.61**_**R**_	**−38.91**	**0.318**	**7.07**_**R**_	**2.08**_**L**_	**−4.99**	**0.392**
	(±10.12)	(±73.97)	(±43.10)		(±25.30)	(±7.26)	(±15.38)	
Flexion Displacement^a^ (°)	**3.28** (±9.83)	**2.06** (±6.29)	**1.22** (±6.74)	**0.513**	**2.18** (±4.31)	**5.65** (±2.58)	**−3.53** (±2.84)	**0.441**
Extension Displacement^a^ (°)	**2.63** (±2.86)	**3.14** (±1.79)	5.77* (±1.95)	**0.038**	**3.78** (±3.47)	**1.85** (±2.09)	**−1.94** (±2.29)	**0.129**
F/E mean Displacement^b^ (°)	**1.80**_**F**_ (±3.58)	**0.46**_**F**_ (±4.51)	**4.34** (±3.32)	**0.462**	**0.49**_**E**_ (±2.93)	**0.06**_**F**_ (±3.41)	**−0.54** (±2.39)	**0.295**
Right inclination Displacement^b^ (°)	**4.55** (±6.75)	**8.12** (±2.94)	**−3.57** (±4.25)	**0.378**	**14.93** (±6.20)	**8.46** (±5.13)	**6.48** (±4.65)	**0.221**
Left inclination Displacement^a^ (°)	**7.55** (±2.36)	**−2.25** (±0.59)	−5.30* (±1.41)	**0.083**	**3.86** (±9.60)	**0.45** (±9.32)	**−3.41** (±6.41)	**0.349**
R/L mean inclination Displacement^a^ (°)	**0.45**_**R**_ (±4.22)	**2.45**_**R**_ (±3.59)	**−2.00** (±3.20)	**0.471**	**16.77**_**R**_ (±20.27)	**2.17**_**R**_ (±5.78)	**14.59** (±12.31)	**0.319**
Left rotation Velocity^b^ (°/s)	**44.17** (±26.91)	**37.25** (±14.22)	**6.93** (±17.34)	**0.403**	**41.65** (±18.28)	**38.22** (±25.45)	**3.43** (±16.37)	**0.177**
Right rotation Velocity^b^ (°/s)	**44.46** (±22.68)	**36.06** (±13.48)	**−8.40** (±14.91)	**0.377**	**45.81** (±17.43)	**41.13** (±22.62)	**−4.68** (15.02)	**0.512**
Flexion Velocity^a^ (°/s)	**30.85** (±15.36)	**32.56** (±20.36)	**−1.71** (±13.39)	**0.279**	**30.69** (±23.91)	**41.72** (±25.30)	**−11.04** (±18.69)	**0.451**
Extension Velocity^a^ (°/s)	**31.78** (±22.38)	**36.72** (±25.62)	**4.94** (±18.12)	**0.227**	**21.86** (±18.44)	**34.74** (±34.19)	**12.88** (±19.79)	**0.286**
Right inclination Velocity^b^ (°/s)	**37.75** (±19.90)	**36.52** (±24.97)	**1.22** (±16.85)	**0.186**	**25.07** (±9.32)	**41.06** (±22.51)	**−15.99** (±12.19)	**0.313**
Left inclination Velocity^b^ (°/s)	**30.2** (±13.70)	**27.73** (±16.14)	**−2.47** (±11.25	**0.036**	**41.68** (±51.46)	**61.69** (±25.59)	**21.02** (±32.82)	**0.315**
Resultant Velocity (°/s) Right Side^a^	**66.37** (±33.96)	**63.13** (±30.55)	**3.24** (±24.93)	**0.483**	**60.21** (±23.76)	**77.77** (±7.40)	**−17.56** (±14.51)	**0.229**
Resultant Velocity (°/s) Left Side^a^	**62.28** (±28.37)	**61.71** (±22.64)	**0.57** (±20.03)	**0.384**	**69.67** (±50.68)	**88.76** (±23.97)	**−19.09** (±18.50	**0.293**

^a^:parametric variables.

^b^:non-parametric variables.

Bold: main values of the variables.

**Table 3:** **Mean values of the kinematic variables during one single leg stance test (eyes closed)**

	Non-affected leg				Affected leg			
	Lumbar (sd)	Trunk (sd)	Difference (sd)	p value	Lumbar (sd)	Trunk (sd)	Difference (sd)	p value
Left rotation Displacement^a^ (°)	**24.7** (±15.15)	**12.56** (±2.61)	**12.15** (±9.05)	**0.364**	**22.66** (±11.42)	**25.19** (±20.27)	**−2.53** (±13.66)	**0.271**
Right rotation Displacement^a^ (°)	**1.07** (±5.27)	**−2.75** (±3.95)	**3.82** (±3.66)	**0.198**	**24.39** (±7.73)	**18.55** (±15.54)	**−5.84** (±10.02)	**0.156**
Rotation Mean Displacement^a^ (°)	**13.61**_**R**_ (±9.87)	**9.89**_**R**_ (±5.58)	**3.73** (±6.43)	**0.252**	**2.71**_**L**_ (±8.84)	**3.58**_**R**_ (±4.37)	**−6.29 (±6.97)**	**0.304**
Flexion Displacement^a^ (°)	**10.21** (±2.24)	**11.56** (±4.50)	**−1.35** (±2.55)	**0.235**	**6.40** (±2.39)	**12.56** (±5.69)	**−6.16** (±3.49)	**0.227**
Extension Displacement^b^ (°)	**1.73** (±4.04)	**0.58** (±2.90)	**1.15** (±2.77)	**0.185**	**6.38** (±2.30)	**5.47** (±0.88)	**−0.91** (±1.47)	**0.241**
F/E mean Displacement^b^ (°)	**7.19**_**F**_ (±1.08)	**5.16**_**F**_ (±2.37)	**2.03** (±1.31)	**0.401**	**1.10**_**F**_ (±1.53)	**2.87**_**F**_ (±2.83)	**−1.77** (±1.88)	**0.463**
Right inclination Displacement^a^ (°)	**3.42** (±4.16)	**1.98** (±3.79)	**1.44** (±3.07)	**0.311**	**19.29** (±8.39)	**15.73** (±4.17)	**3.56** (±5.67)	**0.315**
Left inclination Displacement^a^ (°)	**13.52** (±12.29)	**10.36** (±8.02)	**−3.16** (±8.24)	**0.106**	**18.23** (±20.76)	**22.63** (±22.44)	**4.40** (±19.48)	**0.085**
R/L mean inclination Displacement^b^ (°)	**5.01**_**L**_ (±7.32)	**7.19**_**L**_ (±6.24)	**2.18** (±5.28)	**0.245**	**3.56**_**R**_ (±7.05)	**4.96**_**L**_ (±16.31)	**8.52** (±10.07)	**0.144**
Left rotation Velocity^a^ (°/s)	**45.57** (±20.90)	**50.93** (±20.39)	**−5.37** (±15.81)	**0.315**	**24.27** (±8.80)	**20.91** (±1.33)	**3.36** (±6.60)	**0.217**
Right rotation Velocity^a^ (°/s)	**34.81** (±8.16)	**36.69** (±10.84)	**1.88** (±7.12)	**0.315**	**47.28** (±43.14)	**30.81** (±11.75)	**−16.47** (±32.74)	**0.279**
Flexion Velocity^b^ (°/s)	**23.89** (±12.31)	**40.81** (±34.54)	**−16.93** (±18.21)	**0.189**	**23.50** (±3.44)	**32.19** (±8.23)	**−8.69** (±5.04)	**0.241**
Extension Velocity^b^ (°/s)	**40.45** (±40.23)	**44.52** (±38.84)	**4.07** (±30.31)	**0.401**	**37.33** (±15.02)	**62.29** (±50.56)	**24.96** (±28.90)	**0.199**
Right inclination Velocity^a^ (°/s)	**54.15** (±65.3)	**50.44** (±52.02)	**3.71** (±46.09)	**0.316**	**49.44** (±23.75)	**48.20** (±23.41)	**1.24** (±21.58)	**0.337**
Left inclination Velocity^a^ (°/s)	**23.61** (±12.57)	**29.42** (±10.58)	**5.81** (±9.02)	**0.176**	**41.79** (±16.00)	**55.83** (±4.50)	**14.04** (±12.16)	**0.294**
Resultant Velocity (°/s) Right Side^b^	**171.84** (±244.1)	**111.04** (±42.30)	**60.80** (±145.85)	**0.092**	**60.85** (±21.84)	**62.16** (±21.97)	**−1.31** (±19.97)	**0.131**
Resultant Velocity (°/s) Left Side^b^	**61.13** (±37.42)	**66.63** (±34.11)	**−5.20** (±27.60)	**0.285**	**76.66** (±39.85)	**93.23** (±34.97)	**−16.56** (±34.96	**0.341**

^a^: parametric variables.

^b^: non-parametric variables.

Bold: main values of the variables.

## Discussion

The objectives of this study were to analyse the reliability of inertial sensors during the parameterisation of SLS tests in stroke survivors and to compare data from two different sensor locations (trunk and lumbar) in terms of the kinematic variables measured in the SLS test. Our hypotheses were partially confirmed; inertial sensors proved to be reliable, instruments for the measurement of kinematic data during the SLS test. However, of the 68 kinematic variables analysed, only two (extension displacement and leftward rotation displacement in the non-affected leg, eyes open condition) showed significant differences between the sensor locations. That result tends to disprove our second hypothesis, that there would be differences in the kinematic data related to the location of the sensor.

Inertial sensors are tools frequently used in clinical practice and research [13]. They are validated tools [27] and easily accessible because they are commonly found in quotidian devices such as smartphones for example [12, 14, 15]. These features increase the potential for their use in clinical research, such as the study presented here [12]. Due to the features of the sensors it could be to identify cut points to implement assessment and monitoring in people with stroke.

Moreover, the measurements made with inertial sensors allowed us to analyse the motion in angular displacement, velocity and acceleration. These results are well-aligned with the clinical need to define balance sub-divisions: stability and sway [28]. The main values of the kinematic variables could be used to define the first balance sub-division (stability), while the peak of the kinematic variables could be useful to estimate the second balance sub-division (sway).

### Differences between sensors

Analysis of the data recorded by sensors in both locations revealed significant location differences in only 2 of 68 measured variables which are: displacement in extension and leftward rotation for the non-affected leg, eyes open condition. one might therefore argue that the exact location of the sensor does not matter for recording kinematic data during the SLS test. These results are consistent with previously published studies [29–31]. The consistency in angular measurement has been observed across several conditions: using an angular displacement sensor to test static balance (present study), semi-static functional testing [29], dynamic movement [30] and for angular velocity, where there was no difference between the stem, lumbar, thorax or foot insole sensors [31].

In different studies consulted, the location of the inertial sensors used to analyse different kinematic variables, has been very uneven, placing sensors in the thorax and foot insoles [31], foot and thigh [30] and lumbar and trunk (present study). However, the consistency in recording kinematic variables is maintained regardless of sensor location.

We therefore conclude that there is no significant difference in kinematic data recorded in the lumbar and trunk regions when recordings are made with a single inertial sensor in either of these two locations for the purposes of the SLS test.

### Reliability

We observed excellent reliability in kinematic parameters measured during the SLS test using inertial sensors placed in the trunk and lumbar regions. The reliability values we obtained are consistent with other studies that used inertial sensors in different locations and tests [12, 17, 32].

Reliability values in this study are consistent with the results in other studies [12], but the detailed analysis revealed some points of interest. Lugade and colleagues [17] reported that the intra-observer reliability of measurements in the standing position was 0.96 (95% CI: 0.86–0.99); a range that is in line with the results presented in the present study, where the maximum displacement value ranged from 0.849 (intra-observer reliability for mean left inclination velocity: trunk sensor), to a maximum value of 0.949 (intra-observer reliability for left inclination: lumbar sensor). However, the reliability values reported by Lugade and colleagues [17] for transition velocity are different from the values observed in the present study (minimum ICC = 0.847 for inter-observer reliability in left inclination: trunk sensor). One explanation for the discrepancy between these findings related to the characteristics of the test used. The SLS test required the subject to maintain balance, thus variations in movement and velocity are constrained by this requirement which narrows the range of these variables considerably; whereas Lugade and colleagues analysed movements in various conditions (walking, jogging, stair-climbing, recumbent).

Although both reliability (intra-observer and inter-observer) was excellent for both displacement and velocity, there was a decline in reliability of velocity with respect to displacement, with a maximum displacement reliability of 0.949 (intra-observer reliability in left rotation: lumbar sensor) compared to 0.847 (inter-observer reliability in left inclination: trunk sensor) for velocity. Our data about the reliability of displacement measurements are consistent with previous findings [32]. In this study, velocity reliability was higher than displacement reliability (0.985 vs. 0.90), whereas in our study the reliabilities were similar. In the present study the lowest reliability was observed for inter-observer reliability in left inclination at the trunk sensor (0.138).

The reliability values observed in this study demonstrate that inertial sensors are suitable for the measurement of kinematic variables, such as velocity and angular displacement, in a static equilibrium test. These results complement those published from a previous study [33], which reported that inertial sensors reliably measured kinematic parameters during well-defined gestures (left and right trunk rotation; flexion and extension; inclination), which required the subject to maintain a semi-static balance position.

### Strengths and weaknesses

One of the strengths of this study is that it provides the first report of instrumental measurement in the SLS test, a balance test widely used in clinical practice. Stroke patients were a suitable population in which to assess the sensitivity of inertial sensors in this type of functional test, as this population is known to display balance impairments. Replication of these findings with a larger sample would confirm our results.

## Conclusions

Clinicians and researchers criticize measuring balance from above the centre of gravity. The data obtained in the present study suggests that measuring at thoracic level is consistent with measuring at lumbar, so to measure kinematic variables during SLS testing, the location of the IS between trunk and lumbar region is not relevant. In addition, the present study shows that inertial sensors have the potential to be reliable, valid and sensitive instruments in the context of the SLS test but further research is needed to consolidate the results presented in this paper.

## Abbreviations

ICC: Interclass correlation
IS: Inertial sensors
SLS: Single-leg stance.
